# Constipation—Silicea

**Published:** 1881-02

**Authors:** E. B. Nash

**Affiliations:** Cortland, N. Y.


### CHRONIC CONSTIPATION.—SILICEA.

BY E. B. NASH, M.D., CORTLAND, N. Y.

Aug. 14th, 1879. M. D., young lady aged twenty-five, single, dark hair and eyes, rather tall and thin. Symptoms: constipation of many years' standing, can hardly remember when she was not so. Has taken much cathartic medicine; can not have stool without cathartic medicine or an enema. This enema now fails to procure a passage and causes a sick headache. Appetite good except when she takes her cathartic pills (Herricks), of which she has to take as many as four at once. Then she loses her appetite and has a weak, gone feeling in stomach, with soreness. When she tried to have a passage without her cathartics (as she is inclined to do) "*it comes to the verge of the annus and then recedes as though she* had not the strength *to expel it. The constipation is always worse the week before her menses and she is cold, particularly hands and feet, during that time. Cold sweat of feet sometimes offensive.* Chills run up her legs. Constipation temporarily improved *during* menses.

Has had, for a long time, attacks of sick headache, not at menses. Headaches generally come on between 10–11 A. M., pain *begginning in baek and nape of neck* and extends over to forehead, where it is very severe, is accompanied with nausea, sour and bitter vomiting. Enemas bring this on her. Menses, regular, painful and scanty, which is growing worse. Patient is reduced in flesh, feels discouraged; says she will never be well again. Has tried cathartic medicines, also dieting, and no medicine, but all these failed her and she continues to get worse.

Prescribed *silicea* 200, a powder dry on tongue, two nights in succession, then omit two, etc. Latter part of August she wrote me, "am much better in every way, have a natural stool every day now, and believe you will cure me. Stomach seems sore, food hurts me as it passes through the œsophagus." Continued silicea at longer intervals.

Sept. 17th, has had only one headache since beginning the treatment; natural stools every day except the first few days. Has now been out of medicine some time; for the last week has had a slight return of the constipation, yet stomach is better and food causes no more pain, menses are freer and easier; hands and feet warmer; back improved since first prescription; gained in weight. Prescribed the *silicea* 50 M. (Swan).

This patient remained well up to Feb. 1880, when she came to office on account of a slight gastric trouble.

Comments: I do not know how to classify this disease, pathologically. I treated the patient's symptoms. It was a chronic case, in which allopathic medicine, dieting, etc. were tried and *failed;* I gave nothing below *silicea* 200th, yet my patient recovered, notwithstanding the microscope, the spectroscope, chemistry, etc. can detect nothing therein.

